# Comparative Analysis of Stroke Patients with and without Sequelae: A Cross-Sectional Analysis Using the KOREA National Health and Nutrition Examination Survey (2016–2019)

**DOI:** 10.3390/jcm10184122

**Published:** 2021-09-13

**Authors:** Chi Woong Oh, Sang Hyuk Lee, Taek Min Nam, Ji Hwan Jang, Young Zoon Kim, Kyu Hong Kim, Do-Hyung Kim, Nak Gyeong Ko, Seung Hwan Kim

**Affiliations:** 1 Department of Neurosurgery, Samsung Changwon Hospital, Sungkyunkwan University School of Medicine, Changwon 51353, Korea; ohchiung@naver.com (C.W.O.); shlee858@naver.com (S.H.L.); taekmin82@gmail.com (T.M.N.); gebassist@naver.com (J.H.J.); yzkim@skku.edu (Y.Z.K.); Unikkh@unitel.co.kr (K.H.K.); 2 Department of Neurology, Samsung Changwon Hospital, Sungkyunkwan University School of Medicine, Changwon 51353, Korea; pons1217@gmail.com; 3 Department of Biostatistics, Samsung Changwon Hospital, Sungkyunkwan University School of Medicine, Changwon 51353, Korea; nakgyeong.ko@samsung.com

**Keywords:** stroke, sequelae, sequelae of stroke, sociodemographic factors, depression, suicide

## Abstract

(1) Background: We aimed to evaluate the association between sociodemographic factors and mental health problems and the sequelae of stroke in South Korea by analyzing the annual Korea National Health and Nutrition Examination Surveys (KNHANES) conducted from 2016 to 2019. (2) Methods: Data were obtained from 32,379 participants who participated in the KNHANES (2016–2019). A total of 567 participants diagnosed with stroke were included in this study. Patients were divided into two groups based on the presence of sequelae: (a) stroke patients with sequelae (*n* = 227, 40.0%) and (b) stroke patients without sequelae (*n* = 340, 60.0%). (3) Results: Compared to stroke patients without sequelae, those with sequelae were significantly associated with sex (male, 61.2% vs. 47.6%, *p* = 0.002), household income (lower half, 78.9% vs. 67.4%, *p* = 0.005), owning a house (60.4% vs. 68.5%, *p* = 0.048), marital status (unmarried, 7.05% vs. 1.76%, *p* < 0.001), depression (13.2% vs. 7.35%, *p* = 0.045), suicidal ideation (6.17% vs. 3.24%, *p* = 0.010), and suicide attempts (2.64% vs. 0.88%, *p* = 0.012). (4) Conclusions: Our study showed that poor sociodemographic factors and mental health problems were significantly associated with sequelae from stroke. Clinical physicians should therefore carefully screen for depression and suicidality in stroke patients with sequelae, especially in those with poor sociodemographic factors.

## 1. Introduction

Stroke is a major cause of acquired physical disability in adults worldwide [1,2]. Sequelae from stroke generally include motor weakness and cognitive impairment [1,2]. Stroke patients with sequelae may suffer from mental health problems, such as depression, suicidal ideation, and suicide attempt [3,4,5,6,7,8,9].

Mental health concerns for stroke patients have frequently been reported [5,9,10]. Previous studies have found that stroke patients have higher rates of depression and suicidal ideation, and stroke survivors with depression appear to have a higher mortality rate than those without depression [5]. Another study reported that stroke patients living alone, with low education, and low income had a higher risk of suicide attempts [6,8,10].

This nationwide database analysis study using the Korea National Health and Nutrition Examination Survey (KNHANES) aimed to comprehensively evaluate the association between sociodemographic factors and mental health and stroke sequelae. The KNHANES, which includes not only health status but also sociodemographic and economic factors, is an annual cross-sectional survey designed to examine the health and nutritional status of the Korean population [11].

We hypothesized that stroke patients with sequelae were more likely to have poor sociodemographic factors and to suffer from mental health problems than those without sequelae. We aimed to compare sociodemographic factors and mental health between stroke patients with and without sequelae. We also investigated the factors affecting depression in patients with stroke.

## 2. Materials and Methods

### 2.1. Study Population

This study was based on data obtained from the 2016–2019 KNHANES. During the study period, 32,379 individuals participated in the KNHANES. Among them, the 569 respondents who answered “yes” to the question “Have you ever been diagnosed with stroke by a clinical physician?” were included in this study. The participants were then divided into two groups based on the question “Are you suffering from stroke?”. Individuals who responded “yes” were classified as stroke patients with sequelae (*n* = 227), and individuals who responded “no” were classified as stroke patients without sequelae (*n* = 340).

### 2.2. Variables

The KNHANES includes well-established questions regarding sociodemographic factors, such as sex, age, height, weight, body mass index (BMI), monthly household income, number of household members, house ownership, employment status, marital status, level of education, smoking status, and alcohol consumption. None of the 569 respondents were excluded, even though many had missing data. Due to the considerable number of missing variables, it would have been impossible to perform chi-square tests if all were excluded. However, there were no differences in general characteristics between those who provided complete responses and those who did not.

BMI was calculated by dividing weight (kg) by height (m^2^). In the KNHANES, monthly household income is classified into quartiles (1: low, 2 and 3: middle, and 4: high); however, in this study, it was divided into two groups (lower half and upper half). Employment status was divided into either employed or unemployed. For marital status, participants were categorized as married or unmarried, and those who were widowed or divorced were included in the married group. For level of education, participants were classified into elementary school or lower, middle school, high school, or college or higher. Smoking status was divided into two categories: smokers and non-smokers. Alcohol consumption was divided into two categories: drinkers and non-drinkers.

Three dimensions of mental health were investigated: depression, suicidal ideation, and suicide attempts. Respondents who had been diagnosed with depression by a clinical physician were considered to have depression. Suicidal ideation was evaluated using the response to the following question: “Have you thought about committing suicide in the past year?” (yes or no). Suicide attempt was evaluated using the response to the question: “Have you attempted suicide in the past year?” (yes or no).

### 2.3. Statistical Analysis

All statistical analyses were conducted using SPSS version 22 (IBM Corp., Armonk, NY, USA). The KNHANES data were collected through a representative, stratified, and clustered sampling method. Sociodemographic factors and mental health were compared between stroke patients with and without sequelae, and between stroke patients with and without depression. Categorical variables were analyzed using the chi-square test or Fisher’s exact test. Continuous variables were analyzed using the Student’s *t*-test or the Mann–Whitney U test. Statistical significance was set at *p* < 0.05. The collinearity diagnostic check was performed by calculating the variance inflation factors for all independent variables. All variables had a variance factor value of less than 2, indicating that they were not closely correlated. No adjustment was made for multiplicity. A multivariable logistic regression analysis was conducted to evaluate the factors affecting depression in patients with stroke. All variables with a *p*-value < 0.20 in the univariate analysis were included in the logistic regression analysis.

## 3. Results

Table 1 shows the general characteristics of the participants diagnosed with stroke during the survey period (2016–2019). A total of 567 subjects from the KNHANES (2016–2019) were included in this study. Slightly over half (53.1%) were male, and the mean age was 65.7 ± 11.9 years. More participants had a low monthly household income (score of 1) than a high income (score of 4) (255/567 (45.0%) vs. 61/567 (10.7%)). Most of the participants were married (96.1%), and depression was observed in 55 of the participants (9.70%).

The characteristics of stroke patients with and without sequelae in the KNHANES (2016–2019) are compared in Table 2. In the group of stroke patients with sequelae, 61.2% (139/227) were male, while 47.6% (162/340) were male in the group without sequelae (*p* = 0.002). Additionally, more of the patients with sequelae had monthly household incomes in the lower half than those without sequelae (179/227 (78.9%) vs. 229/340 (67.4%), p = 0.005). Fewer of the stroke patients with sequelae owned a house than those without sequelae (137/227 (60.4%) vs. 232/340 (68.2%), *p* = 0.048). Furthermore, there were more unmarried individuals in the group of stroke patients with sequelae than in the group without sequelae (16/227 (7.05%) vs. 6/340 (1.76%), *p* < 0.001). Lastly, stroke patients with sequelae were more likely to be smokers than those without sequelae (126/227 (55.5%) vs. 154/340 (45.3%), *p* = 0.016).

Stroke patients with sequelae had poorer mental health, including depression, suicidal ideation, and suicide attempts, than those without sequelae. Depression was observed more frequently in stroke patients with sequelae than in those without sequelae (30/227 (13.2%) vs. 25/340 (7.35%), *p* = 0.045). Suicidal ideation and suicide attempts were also observed more frequently in stroke patients with sequelae than in those without sequelae (14/227 (6.17%) vs. 11/340 (3.24%), *p* = 0.010, and 6/227 (2.64%) vs. 3/340 (0.88%), *p* = 0.012, respectively).

In Table 3, the characteristics of stroke patients with and without depression in the KNHANES (2016–2019) are compared. There were fewer males in the group of stroke patients with depression than in the group without depression (15/55 (27.3%) vs. 286/512 (55.9%), *p* < 0.001). Stroke patients with depression had a lower employment rate than those without depression (8/55 (15.5%) vs. 182/512 (35.5%), *p* < 0.001). Additionally, smokers were observed more frequently in stroke patients without depression than in those with depression (263/512 (51.4%) vs. 17/55 (30.9%), *p* = 0.002). Lastly, suicidal ideation and suicide attempts were observed more frequently in stroke patients with depression than in those without depression (7/55 (12.7%) vs. 18/512 (3.52%), *p* = 0.003, and 6/55 (10.9%) vs. 3/512 (0.59%), *p* < 0.001, respectively).

The multivariable analysis of factors related to depression in stroke patients, which included sex, height, BMI, house ownership, employment status, smoking status, alcohol consumption, and sequelae of stroke, revealed that sex (female) (adjusted odds ratios (OR): 3.878, adjusted 95% confidence interval (CI): 2.060–7.301, *p* < 0.001), and presence of stroke sequelae (adjusted OR: 2.443, adjusted 95% CI: 1.368–4.364, *p* = 0.003) were independently associated with depression in stroke patients (Table 4).

## 4. Discussion

In this nationwide population-based study of stroke patients, we observed sociodemographic differences between stroke patients with and without sequelae. Our results showed that stroke patients with sequelae had poorer sociodemographic factors, such as lower monthly household income, not owning a house, and not being married, than those without sequelae. This study suggests that stroke patients with sequelae have a poorer sociodemographic status than those without sequelae. Our results also showed that the percentage of stroke patients with a monthly household income of 1 (low) was close to 50%, suggesting that stroke patients tended to have relatively low incomes.

Sociodemographic factors may have an influence on the sequelae after stroke because socially disadvantaged patients are less likely to receive appropriate treatments [12,13,14]. The presence of sequelae after stroke may also have an influence on patients’ socioeconomic status [3,13,14]. Since this was a cross-sectional study, no causal conclusions could be drawn. However, to the best of our knowledge, this study is the first to compare sociodemographic factors between stroke patients with and without sequelae using a nationwide survey.

Previous studies have reported that stroke patients have higher rates of depression and suicidal ideation [3,5,6,10]. In our study, we considered sociodemographic factors and mental health problems in stroke patients with and without sequelae using nationwide data and found that stroke patients with sequelae had more mental health issues than those without sequelae. This can therefore be considered a foundational study of the association between sociodemographic factors and mental health problems and the presence of sequelae in stroke patients. 

In this study, the proportion of stroke patients diagnosed with depression was 9.70%. Our results also showed that stroke patients with depression had higher rates of sequelae and a lower rate of employment than those without depression. Depression could therefore be linked to physical disability and subsequent unemployment resulting from stroke [9,15,16].

Female sex is known to be an important risk factor for depression in the general population [17]. Previous studies on depression in stroke patients conducted in other countries have reported that the rate of depression does not differ significantly between male and female stroke patients [18]; however, our study showed that female sex was independently associated with depression in stroke patients. This can be partially explained by international and cultural differences among countries.

In this study, a strong association between depression and stroke sequelae was observed. Previous studies have reported that the loss of physical function and independence are strongly associated with depression and suicidal ideation in stroke patients [9,16], which is consistent with our results. This study emphasized the importance of carefully screening for depression and suicidality in stroke patients, especially in those with sequelae and poor sociodemographic factors.

This study also has several limitations. First, knowing the temporal relationship between stroke and mental health problems would be helpful to understand the effect of mental health problems on stroke outcomes and the effect of stroke outcomes on mental health problems. However, given that this was a cross-sectional analysis, it was impossible to draw causal conclusions. Second, although the study included a large sample from a nationwide health survey, most of the data were self-reported. The potential issues associated with self-reported data are label errors, recall bias, and a lack of accuracy. Third, knowing the stroke severity, types of stroke (such as hemorrhagic stroke and ischemic stroke), and specific types of sequelae of stroke (such as hemiparesis, sensory change, aphasia, and cognitive dysfunction) can give us a better understanding of mental health problems after stroke as they relate to such factors. However, KNHANES did not have data on the stroke severity, types of stroke, and types of sequelae of stroke. Only individual-level data on the presence or absence of stroke and sequelae were included, which was a limitation of this study. Fourth, there may be misclassification bias in the classification of this study, such as patients with and without stroke, sequelae of stroke, and mental health problems. Validation studies are needed to estimate the reliability of this classification. Further epidemiologic studies are needed to determine the differences in sociodemographic factors and mental health between stroke patients with and without sequelae and to assess international and cultural differences.

## 5. Conclusions

Using a large population-based survey, we observed that stroke patients with sequelae had poorer sociodemographic factors and more mental health problems than those without sequelae. We also found that the risk of depression in stroke patients was related to female sex and the presence of sequelae. Our findings suggest that clinicians should carefully screen for depression and suicidality in stroke patients with sequelae, especially in those with poor sociodemographic factors.

## Figures and Tables

**Table 1:** General characteristics of the study subjects retrieved from the Korea National Health and Nutrition Examination Survey (KNHANES) (2016–2019).

Variables		Stroke patients (*n* = 567)
Sociodemographic factors	Sex (male)	301 (53.1%)
	Age (years)	65.7 ± 11.9
	Height (cm)	161.2 ± 9.07
	Weight (kg)	64.1 ± 11.7
	Body mass index (kg/m^2^)	24.6 ± 3.34
	Monthly household income	
	1 (low)	255 (45.0%)
	2 (middle)	153 (27.0%)
	3 (middle)	98 (17.3%)
	4 (high)	61 (10.7%)
	Number of household members	
	1	128 (22.6%)
	2	254 (44.8%)
	3	102 (18.0%)
	4	43 (7.58%)
	≥5	40 (7.05%)
	House ownership	369 (65.1%)
	Employment status (employed)	190 (33.5%)
	Marital status (married)	545 (96.1%)
	Level of education	
	Elementary school or lower	285 (50.3%)
	Middle school	98 (17.3%)
	High school	123 (21.7%)
	College or higher	61 (10.8%)
	Smoking status (smoker)	280 (49.4%)
	Alcohol consumption (drinker)	455 (80.2%)
Mental health	Depression	55 (9.70%)
	Suicidal ideation	25 (4.41%)
	Suicide attempts	9 (1.59%)

**Table 2:** A comparison of stroke patients with and without sequelae retrieved from the Korea National Health and Nutrition Examination Survey (KNHANES) (2016–2019).

Variables		Sequelae of Stroke		*p*-Value
		Present (*n* = 227)	Absent (*n* = 340)	
Sociodemographic factors	Sex (male)	139 (61.2%)	162 (47.6%)	0.002
	Age (years)	64.2 ± 12.6	66.7 ± 11.2	0.468
	Height (cm)	161.1 ± 9.22	159.5 ± 8.85	0.040
	Weight (kg)	63.7 ± 11.8	63.1 ± 11.0	0.551
	Body mass index (kg/m^2^)	24.4 ± 3.28	24.8 ± 3.48	0.279
	Monthly household income			0.005
	Lower half	179	229	
	Upper half	48	111	
	Number of household members			0.500
	1	54	74	
	2	106	148	
	3	35	67	
	4	18	25	
	≥5	14	26	
	House ownership	137 (60.4%)	233 (68.5%)	0.048
	Employment status (employed)	65 (28.6%)	125 (36.8%)	0.151
	Marital status (unmarried)	16 (7.05%)	6 (1.76%)	<0.001
	Level of education			0.221
	Elementary school or lower	108	177	
	Middle school	35	63	
	High school	57	66	
	College or higher	25	36	
	Smoking status (smoker)	126 (55.5%)	154 (45.3%)	0.016
	Alcohol consumption (drinker)	184 (81.1%)	271 (79.7%)	0.121
Mental health	Depression	30 (13.2%)	25 (7.35%)	0.045
	Suicidal ideation	14 (6.17%)	11 (3.24%)	0.010
	Suicide attempts	6 (2.64%)	3 (0.88%)	0.012

**Table 3:** A comparison of the study subjects with and without depression retrieved from the Korea National Health and Nutrition Examination Survey (KNHANES) (2016–2019).

Variables		Depression		*p*-Value
		Present (*n* = 55)	Absent (*n* = 512)	
Sociodemographic factors	Sex (male)	15 (27.3%)	286 (55.9)	<0.001
	Age (years)	68.0 ± 10.6	68.1 ± 10.8	0.946
	Height (cm)	157.8 ± 9.00	160.4 ± 9.00	0.044
	Weight (kg)	63.0 ± 9.95	63.4 ± 11.5	0.791
	Body mass index (kg/m^2^)	25.3 ± 3.43	24.6 ± 3.40	0.142
	Monthly household income			0.814
	Lower half	41	367	
	Upper half	14	145	
	Number of household members			0.292
	1	20	108	
	2	20	234	
	3	7	95	
	4	2	41	
	≥5	6	34	
	House ownership	30 (54.5%)	340 (66.4%)	0.100
	Employment status (employed)	8 (14.5%)	182 (35.5%)	<0.001
	Marital status (unmarried)	2 (3.6%)	20 (3.9%)	1.000
	Smoking status (smoker)	17 (30.9%)	263 (51.4%)	0.002
	Alcohol consumption (drinker)	39 (70.9%)	416 (81.3%)	0.053
	Sequelae of stroke	30 (54.5%)	197 (38.5%)	0.045
Mental health	Suicidal ideation	7 (12.7%)	18 (3.52%)	0.003
	Suicide attempts	6 (10.9%)	3 (0.59%)	<0.001

**Table 4:** Multivariable logistic regression analysis of the factors affecting depression in stroke patients retrieved from the Korea National Health and Nutrition Examination Survey (KNHANES) (2016–2019).

Factors	Adjusted OR	Adjusted 95% CI	*p*-Value
Sex (female)	3.878	2.060–7.301	<0.001
Sequelae of stroke	2.443	1.368–4.364	0.003

OR: Odds ratio, CI: Confidence interval.
